# Supplementary material for: GRF2 Is Crucial for Cone Photoreceptor Viability and Ribbon Synapse Formation in the Mouse Retina
Source: Cells. 2023 Nov 4;12(21):2574. doi: 10.3390/cells12212574 (PMC10650203; doi:10.3390/cells12212574)
Supplement: Supplementary file 1 [file cells-12-02574-s001.zip › Figure legend S1.docx]

Figure S1. GRF2 expression in photoreceptors and Müller cells. A) Co-localization of the GRF2 BaseScope probe signal with PNA immunostaining in cone photoreceptors (arrows). Scale bar 10μm. B) GLAST immunostaining and GRF2 ISH signal in the Müller glial cells. Scale bar 20μm.
