# Supplementary figures and images for: GRF2 Is Crucial for Cone Photoreceptor Viability and Ribbon Synapse Formation in the Mouse Retina
Source: Cells. 2023 Nov 4;12(21):2574. doi: 10.3390/cells12212574 (PMC10650203; doi:10.3390/cells12212574)

A

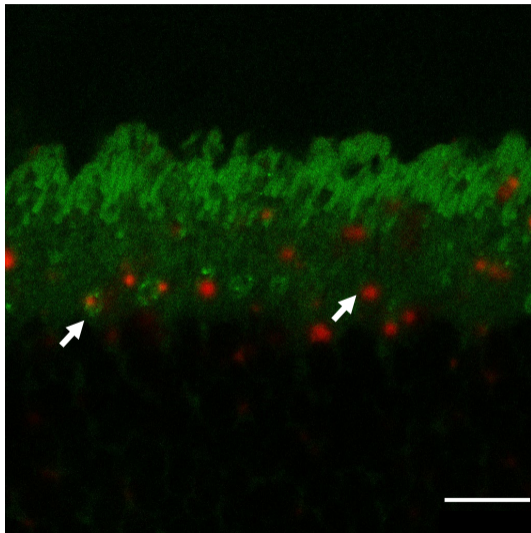

B

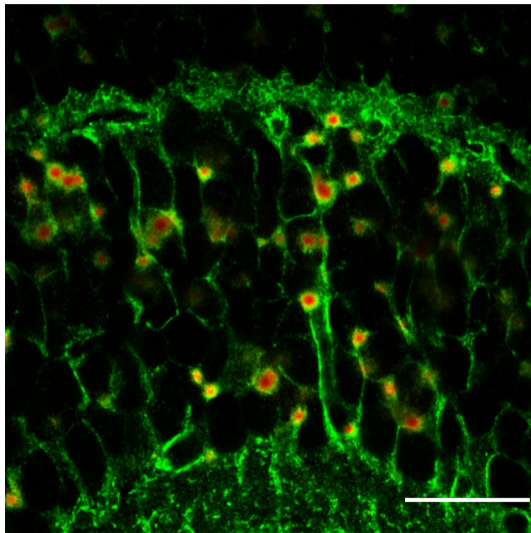

Supplement: Supplementary file 1 [file cells-12-02574-s001.zip › Figure S1 last.pdf]
